# Amiselimod (MT-1303), a Novel Sphingosine 1-Phosphate Receptor-1 Modulator, Potently Inhibits the Progression of Lupus Nephritis in Two Murine SLE Models

**DOI:** 10.1155/2019/5821589

**Published:** 2019-12-23

**Authors:** Kunio Sugahara, Yasuhiro Maeda, Kyoko Shimano, Mikako Murase, Sachiko Mochiduki, Kana Takemoto, Tetsuhiro Kakimoto, Hiroyuki Utsumi, Koichi Oshita, Hirotoshi Kataoka

**Affiliations:** Sohyaku, Innovative Research Division, Mitsubishi Tanabe Pharma Corporation, Yokohama 227-0033, Japan

## Abstract

Amiselimod (MT-1303) is a novel and selective sphingosine 1-phosphate receptor-1 (S1P_1_) modulator with a more favorable cardiac safety profile than other S1P_1_ receptor modulators. In this study, we evaluated the effects of MT-1303 on the progression of lupus nephritis in two well-known murine systemic lupus erythematosus (SLE) models, MRL/*lpr* and NZBWF1 mice, compared with those of FK506. Daily oral doses of 0.1 and 0.3 mg/kg MT-1303 not only inhibited the development of lupus nephritis when administered before onset in MRL/*lpr* and NZBWF1 mice but also improved symptoms of lupus nephritis when administered after onset in MRL/*lpr* mice. Its efficacy in these models was more potent or comparable to that of FK506 (1 and 3 mg/kg). In histological analysis, treatment with MT-1303 inhibited infiltration of T cells into the kidneys, mesangial expansion, and glomerular sclerosis. MT-1303 treatment resulted in a marked reduction in T cells and B cells in the peripheral blood and significantly inhibited increases in the number of plasma cells in the spleen and T cells in the kidneys. In addition, administration of MT-1303 suppressed elevations in serum anti-dsDNA antibody levels in MRL/*lpr* mice, but not in NZBWF1 mice. Our findings show that MT-1303 exhibits marked therapeutic effects on lupus nephritis in two SLE models, likely by reducing the infiltration of autoreactive T cells into the kidneys. These results suggest that MT-1303 has the potential to be used as a therapeutic agent for patients suffering from SLE, including lupus nephritis.

## 1. Introduction

Amiselimod (MT-1303) is an oral selective sphingosine 1-phosphate receptor-1 (S1P_1_) modulator [1] that is currently being developed for the treatment of various autoimmune diseases. A phase I study demonstrated that MT-1303 has a more favorable cardiac safety profile than fingolimod, the first S1P_1_ receptor modulator approved for the treatment of relapsing-remitting multiple sclerosis (RRMS) [2]. A phase II study that enrolled more than 400 patients with RRMS reported that MT-1303 at doses up to 0.4 mg had superior efficacy over the placebo control as well as a benign safety profile [3]. MT-1303 is converted to its active metabolite, MT-1303 phosphate (MT-1303-P), and acts as a functional antagonist of the S1P_1_ receptor. MT-1303-P induces S1P_1_ receptor internalization in lymphocytes, inhibits lymphocyte egress from secondary lymphoid organs by reducing the S1P responsiveness of lymphocytes, and consequently exerts immunomodulatory activity by markedly reducing the number of circulating lymphocytes [1].

Systemic lupus erythematosus (SLE) is a chronic autoimmune disease characterized by the production of a wide variety of autoantibodies and immune complex-mediated tissue inflammation [4–7]. Patients with SLE are susceptible to organ damage accrual caused by both active disease and medication toxicities [8, 9]. Development of an effective treatment with an acceptable safety profile, particularly one with high disease activity, and corticosteroid reduction is warranted [10, 11]. Belimumab, which inhibits the B cell activating factor (BAFF), a key survival cytokine for B cells, is currently the only approved biological agent for SLE [12]. However, the efficacy of drugs targeting B cells is limited, and other approaches, including those targeting T cells, are required to improve treatment options for SLE patients [13, 14].

S1P_1_ receptor modulators suppress infiltration of autoreactive T cells into sites of inflammation by inhibiting lymphocyte egress from secondary lymphoid organs [15] and have proven their therapeutic potential in RRMS and ulcerative colitis [3, 16, 17]. In addition, fingolimod reportedly reduces the production of high-affinity, class-switched antibodies by reducing the formation of the germinal center in the T cell-dependent antibody formation system in mice [18]. Therefore, S1P_1_ receptor modulators are expected to have therapeutic potential against SLE by inhibiting infiltration of autoreactive T cells into sites of inflammation and by affecting autoantibody production. In fact, studies have reported that S1P_1_ receptor modulators are efficacious in reducing proteinuria and improving kidney histology in MRL/*lpr*, NZBWF1, and BXSB mouse models of SLE [19–23].

In the present study, we evaluated the prophylactic and therapeutic effects of MT-1303 on lupus nephritis progression in MRL/*lpr* and NZBWF1 mice compared with FK506. MRL/*lpr* and NZBWF1 mice, well-known animal models of SLE, develop lupus nephritis spontaneously [24, 25]. In addition, we investigated the effects of MT-1303 on infiltration of T cells into the kidneys and autoantibody production in these mice.

## 2. Materials and Methods

### 2.1. Mice

Male MRL/*lpr* mice and female NZBWF1 mice were purchased from the Shizuoka Laboratory Animal Center (Shizuoka, Japan). Three to five mice were housed per plastic cage under specific pathogen-free conditions. They were kept at a constant temperature of 23 ± 3°C and relative humidity of 30–70% under a 12 h light/dark cycle. Food and water were available *ad libitum*. All animal experiments were performed using experimental protocols approved by the ethics review committee for animal experimentation of the Research Division of Mitsubishi Tanabe Pharma. All experimental procedures were as humane as possible.

### 2.2. Agents and Antibodies

Amiselimod (MT-1303; 2-amino-2-{2-[4-(heptyloxy)-3-(trifluoromethyl)phenyl] ethyl}propan-1,3-diol hydrochloride) was provided by Mitsubishi Tanabe Pharma (Osaka, Japan) and was dissolved in 0.5% hydroxypropylmethyl cellulose (HPMC) solution for oral administration. FK506 (Prograf Injection) was purchased from Astellas Pharma (Tokyo, Japan) and diluted with distilled water. The monoclonal antibodies (mAbs) used were obtained from BD Biosciences (Franklin Lakes, NJ, USA) or eBioscience (San Diego, CA, USA) and comprised the following: anti-CD3 mAb (clones 145-2C11 and 555273), anti-CD4 mAb (GK1.5), anti-CD8 mAb (53-6.7), anti-CD45R/B220 mAb (RA3-6B2), anti-CD38 mAb (90), and anti-CD138 mAb (281-2).

### 2.3. Study Design

#### 2.3.1. MRL/*lpr* Study

To evaluate the prophylactic effect, MRL/*lpr* mice at 8 weeks of age without proteinuria (score of 0 or 1) were divided into 4 groups (*n* = 12 each) using the simulation method so that each group had equal mean and variance of body weight and proteinuria score. MT-1303 (0.1, 0.3, or 1 mg/kg) or vehicle was orally administered to the mice daily for 18 weeks, and the effects on the development of lupus nephritis, splenomegaly, and lymphadenopathy were evaluated. Additionally, MT-1303 (0.1 or 0.3 mg/kg) or vehicle was administered to MRL/*lpr* mice at 8 weeks of age for 12 weeks (*n* = 12 each), and levels of anti-dsDNA Ab in serum (*n* = 12) and plasma cell counts in the spleen (*n* = 4) were determined. To evaluate the therapeutic effect, MRL/*lpr* mice at 14 or 16 weeks of age with a proteinuria score of 2 were pooled, randomized by body weight, and divided into 5 groups (*n* = 16 each). MT-1303 (0.1 or 0.3 mg/kg), FK506 (1 or 3 mg/kg), or vehicle was administered for 6 weeks, and the effect on the progression of lupus nephritis was assessed.

#### 2.3.2. NZBWF1 Study

NZBWF1 mice at 30 weeks of age without proteinuria (score of 0 or 1) were divided into 5 groups (*n* = 12 each). MT-1303 (0.1 or 0.3 mg/kg), FK506 (1 or 0.3 mg/kg), or vehicle was administered for 10 weeks, and the effects on the development of lupus nephritis were evaluated. Histological evaluation of the kidneys was conducted in half of the mice in each group (*n* = 6 each) except for the FK506 3 mg/kg group after the final administration. Additionally, MT-1303 (0.3 mg/kg) or vehicle was administered to NZBWF1 mice at 28 weeks of age for 13 weeks, and infiltration of lymphocytes into the kidneys, plasma cell counts in the spleen, and levels of anti-dsDNA Ab in serum were determined (*n* = 8 each).

### 2.4. Evaluation of Lupus Nephritis

The severity of renal disease was monitored by measuring proteinuria. Proteinuria was assessed once a week using Ames urinalysis strips (Albustix®; Siemens Healthcare Diagnostics, Tarrytown, NY, USA) and scored on a scale of 0–4 based on urinary protein concentrations, as follows: 0, <30 mg/dL; 1, 30–100 mg/dL; 2, 100–300 mg/dL; 3, 300–1000 mg/dL; and 4, >1000 mg/dL. In the NZBWF1 study, for more precise and objective evaluation, urinary protein concentrations were measured with a microplate spectrophotometer (SpectraMax 190; Molecular Devices, Sunnyvale, CA, USA) using the Coomassie Plus (Bradford) Assay Kit (Thermo Fisher Scientific, Waltham, MA, USA) and scored on a scale of 0–4. A proteinuria score of 2 and above was defined as positive for proteinuria. Animals that were euthanized or died due to lupus nephritis progression were assigned a subsequent urinary protein concentration of 10 mg/mL, the maximum value.

### 2.5. Histology and Immunohistochemical Staining

Kidney tissue was fixed in 10% buffered formalin, embedded in paraffin, cut into 3 *μ*m thick sections and stained with hematoxylin and eosin (HE) and periodic acid-Schiff (PAS). Kidney sections were evaluated and scored for multiple morphological features of lupus nephritis (mesangial expansion, glomerular sclerosis, interstitial infiltrates, and perivascular infiltrates) by an independent pathologist. Kidney sections were scored using a 4-point scale: 0, no change; 1, mild; 2, moderate; and 3, severe, and the average score was calculated per animal per group.

For immunohistochemical staining, kidney tissue was fixed in zinc fixative, embedded in paraffin, and cut into 3 *μ*m thick sections. The sections were incubated with rat anti-mouse CD3 mAb (555273) followed by secondary mAb conjugated to amino acid polymer and peroxidase (Histofine®, Nichirei Biosciences, Tokyo, Japan). The colorimetric reaction was performed using diaminobenzidine in the presence of hydrogen peroxide, and sections were counterstained with hematoxylin.

### 2.6. Lymphocyte Analyses

Using the IMMUNOPREP™ Reagent System (Beckman Coulter, Brea, CA, USA), 0.1 mL of peripheral blood was hemolyzed and fixed. The number of lymphocytes, T cells, B cells, CD3/B220 double-positive T cells, CD4 T cells, and CD8 T cells was measured using a Cytomics™ FC500 flow cytometer (Beckman Coulter) with Flow-Count™ (Beckman Coulter) as the internal standard. Single-cell suspensions were prepared from the spleen. Red blood cells were lysed, cell suspensions were reconstituted in phosphate-buffered saline, and the number of plasma cells (CD138^+^B220^low^) was measured using a flow cytometer [26]. Kidneys were minced and digested for 30 min at 37°C with 400 U/mL collagenase IV (Worthington Biochemical, Lakewood, NJ, USA). The kidney cell suspensions were subjected to a Lympholyte M gradient (Cedarlane, Burlington, Ontario, Canada) and spun at 2300 rpm for 20 min. The number of lymphocytes, T cells, B cells, CD4 T cells, CD8 T cells, CD3^+^CD4^−^CD8^−^, and double-negative (DN) T cells was measured.

### 2.7. Anti-dsDNA Antibody

Serum levels of the anti-dsDNA antibody were measured using a mouse anti-dsDNA ELISA kit (Shibayagi, Gunma, Japan).

### 2.8. Statistical Analyses

The results are expressed as the mean ± S.E.M. Statistical differences were analyzed using Student's *t*-test, Williams test, or Shirley-Williams test. Statistical differences in proteinuria incidence were calculated using Fisher's exact test with Hommel's multiple comparison test by comparing with the control. Differences between groups were considered significant at *p* < 0.05.

## 3. Results

### 3.1. MT-1303 Strongly Inhibits the Development of Lupus Nephritis in MRL/*lpr* Mice

**Figure 1:**
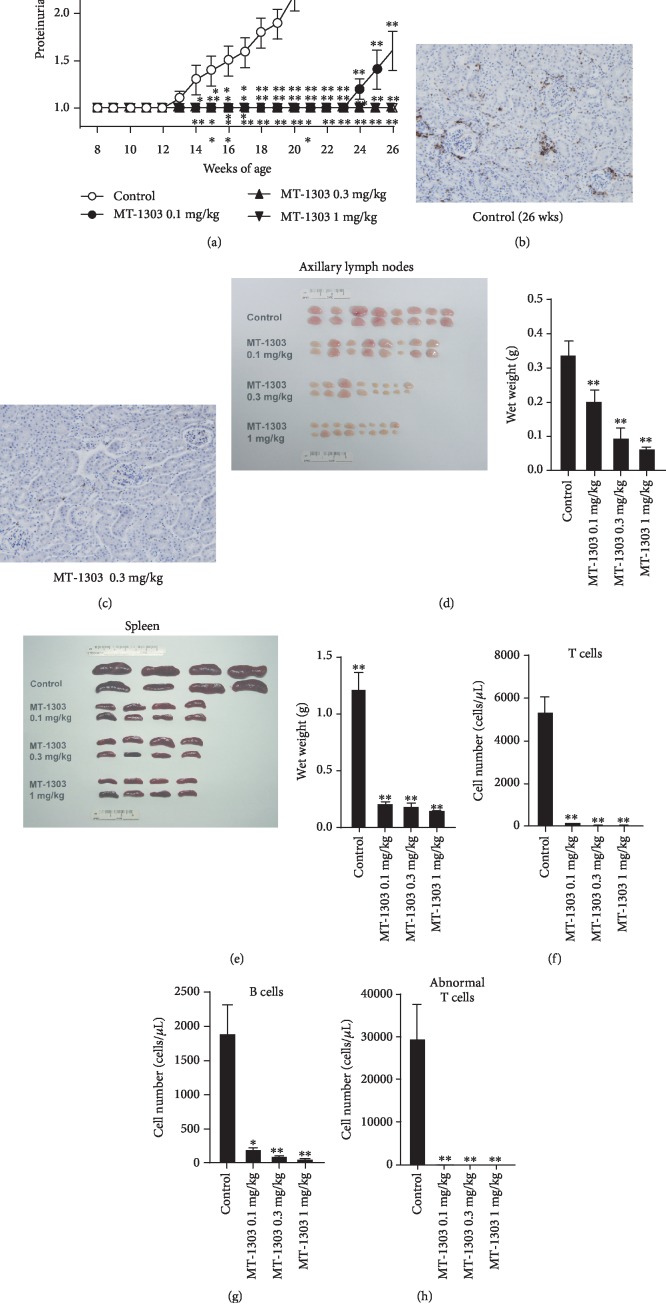
Prophylactic effect of MT-1303 on the development of proteinuria in MRL/*lpr* mice. MT-1303 was administered to MRL/*lpr* mice for 18 weeks from 8 weeks of age. (a) Proteinuria was assessed once a week using Ames urinalysis strips and was scored on a scale of 0–4 based on urinary protein concentrations as described in Materials and Methods. Each symbol represents the mean ± S.E.M. score of 12 animals. Statistical significance was calculated using the Shirley-Williams test by comparison with the vehicle-treated control group (^∗^*p* < 0.05, ^∗∗^*p* < 0.01). (b, c) Kidney sections from vehicle- (b) or MT-1303 0.3 mg/kg- (c) treated mice were labeled with anti-mouse CD3 mAb. (d, e) The axillary lymph nodes (d) and spleen (e) were weighed on the day after final administration. Results are expressed as the mean ± S.E.M. of 8 mice. (f–h) The number of T cells (d), B cells (e), and abnormal T cells (CD3^+^B220^+^) (f) was measured by flow cytometry. Results are expressed as the mean ± S.E.M. of 4 mice. (d–h) Statistical differences were calculated using the Williams test by comparison with the control (^∗^*p* < 0.05, ^∗∗^*p* < 0.01).

To determine the effects of MT-1303 on the development of lupus nephritis, MRL/*lpr* mice at 8 weeks of age were administered vehicle or MT-1303 (oral doses of 0.1, 0.3, and 1 mg/kg) daily for 18 weeks. The time course of changes in proteinuria scores are shown in Figure 1(a). In the vehicle-treated control group, mean proteinuria scores increased with time from 13 weeks of age, and all mice developed lupus nephritis at 21 weeks of age. In contrast, proteinuria scores in all MT-1303-treated groups were significantly lower than those in the control group, and no mice treated with MT-1303 at doses greater than 0.3 mg/kg developed lupus nephritis during the study period. Immunohistochemical staining with anti-CD3 mAb revealed that T cells had infiltrated the kidneys of vehicle-treated control mice at 26 weeks, particularly the periglomeruli area (Figure 1(b)). However, administration of MT-1303 markedly reduced this infiltration of T cells into the kidneys (Figure 1(c)). Moreover, in all MT-1303-treated groups, the weight of the axillary lymph nodes and spleen was significantly lower than that in the control group on the day after the final administration (Figures 1(d) and 1(e)), indicating that MT-1303 also inhibited the development of lymphadenopathy and splenomegaly. To confirm the effects of MT-1303, the number of peripheral blood lymphocytes was measured the day after the final administration (Figures 1(f) and 1(h)). In all MT-1303-treated groups, the number of T cells, CD4 T cells, B cells, and MRL/*lpr*-mouse-specific abnormal T cells was markedly lower than that in the control group.

### 3.2. MT-1303 Suppresses the Production of Anti-dsDNA IgG and Plasma Cells

**Figure 2:**
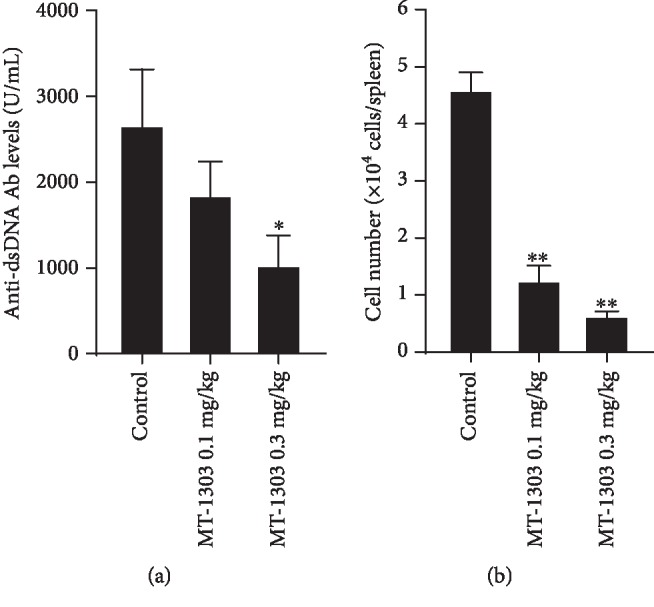
Effect of MT-1303 on anti-DNA antibody levels and plasma cells in MRL/*lpr* mice. MT-1303 was orally administered to MRL/*lpr* mice daily from 8 to 18 weeks of age. The serum and spleen were obtained the day after the final administration, and serum anti-dsDNA antibody levels (a) were determined by ELISA. Results are expressed as the mean ± S.E.M. of 12 mice. The number of plasma cells in the spleen (b) was determined by flow cytometry. Results are expressed as the mean ± S.E.M. of 4 mice. Statistical differences were calculated using the Williams test by comparison with the control (^∗^*p* < 0.05, ^∗∗^*p* < 0.01).

MT-1303 at 0.1 or 0.3 mg/kg was administered to 8-week-old MRL/*lpr* mice for 10 weeks, and serum levels of anti-dsDNA IgG and the number of plasma cells were determined. Treatment with MT-1303 dose-dependently suppressed the increase in levels of anti-dsDNA IgG, with levels in the 0.3 mg/kg MT-1303 group decreasing significantly to around 40% of that in the control group (Figure 2(a)). The number of plasma cells in the spleen was dose-dependently and significantly lower than that in the control group (Figure 2(b)).

### 3.3. MT-1303 Has Therapeutic Effects on Lupus Nephritis in MRL/*lpr* Mice

**Figure 3:**
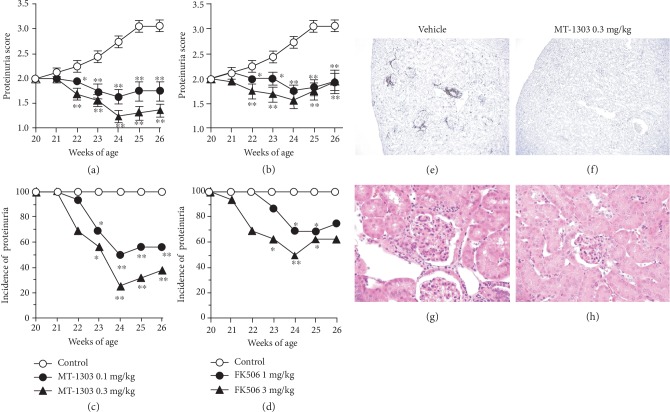
Therapeutic effects of MT-1303 and FK506 on established proteinuria in aged MRL/*lpr* mice. MT-1303 and FK506 were orally administered to MRL/*lpr* mice with a proteinuria score of 2 from 20 to 26 weeks of age. Proteinuria was assessed once a week using Ames urinalysis strips. (a, b) Proteinuria scores following administration of MT-1303 (a) and FK506 (b) are expressed as the mean ± S.E.M. of 16 mice. Statistical differences were calculated using the Shirley-Williams test by comparison with the control (^∗^*p* < 0.05, ^∗∗^*p* < 0.01). (c, d) The incidence of proteinuria in MT-1303 groups (c) and FK506 groups (d) is expressed as the proportion of proteinuria-positive mice (proteinuria score ≥ 2) from a total of 16 mice. Statistical differences were calculated using Fisher's exact test with Hommel's multiple comparison test by comparison with the control (^∗^*p* < 0.05, ^∗∗^*p* < 0.01). (e–h) Kidney sections from vehicle- (e, g) or MT-1303 0.3 mg/kg- (f, h) treated mice were stained with anti-mouse CD3 mAb (e, f) or HE (g, h).

Next, we directly compared the therapeutic effects of MT-1303 and FK506 on lupus nephritis in MRL/*lpr* mice. MT-1303 (0.1 and 0.3 mg/kg) or FK506 (1 and 3 mg/kg) was orally administered to MRL/*lpr* mice with established lupus nephritis for 6 weeks. The time course of changes in proteinuria scores and incidence of proteinuria are shown in Figures 3(a) and 3(b) and 3(c) and 3(d), respectively. In the control group, the mean proteinuria score gradually increased, confirming the progression of lupus nephritis. In contrast, from 2 weeks after the initiation of administration, mean proteinuria scores in all MT-1303-treated groups were significantly lower than those in the control group. In addition, the incidence of lupus nephritis, which was 100% at the initiation of administration, decreased to 56.3% and 37.5% at 6 weeks after administration of 0.1 and 0.3 mg/kg MT-1303, respectively. Additionally, treatment with FK506 significantly inhibited the progression of lupus nephritis, and the therapeutic efficacy of MT-1303 was comparable with that of FK506. Histological analysis of kidney sections showed glomerular lesion, proliferation of mesangial cells, and infiltration of T cells in the control group, while the severity of these lesions was reduced following treatment with MT-1303 (Figures 3(e)–3(h)).

### 3.4. MT-1303 Prevents Progression of Lupus Nephritis in NZBWF1 Mice

**Figure 4:**
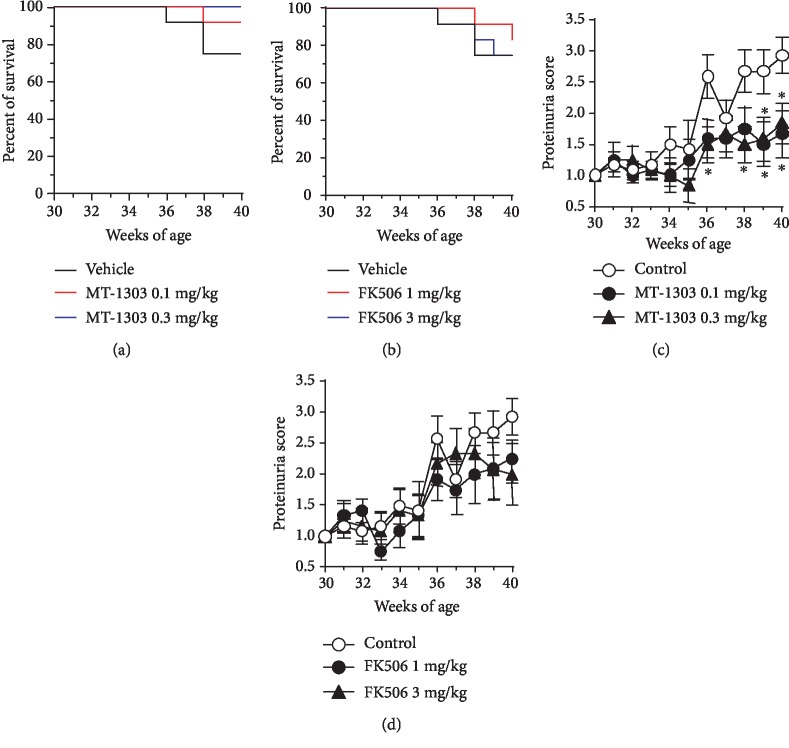
Effects of MT-1303 and FK506 on the development of proteinuria in NZBWF1 mice. MT-1303 and FK506 were orally administered to NZBWF1 mice daily from 30 to 40 weeks of age. (a) Percent survival in each group was shown over the study period. (b, c) Urinary protein concentrations were measured using the Bradford method once a week and scored on a scale of 0–4. Results are expressed as the mean ± S.E.M. of 12 mice. Statistical differences were calculated using the Shirley-Williams test by comparison with the control (^∗^*p* < 0.05).

Thirty-week-old female NZBWF1 mice, divided into 5 groups according to their proteinuria scores and body weight, were orally treated with vehicle, MT-1303 (0.1 and 0.3 mg/kg), or FK506 (1 and 3 mg/kg) daily for 10 weeks until 40 weeks of age, and the effects of these drugs on lupus nephritis were evaluated. Survival rates during the study are shown in Figures 4(a) and 4(b). The number of animals that were euthanized or died due to worsening conditions from lupus nephritis progression during the observation period was 3, 1, 0, 2, and 3 in the vehicle control, MT-1303 0.1 mg/kg-treated, MT-1303 0.3 mg/kg-treated, FK506 1 mg/kg-treated, and FK506 3 mg/kg-treated groups, respectively. In the vehicle-treated control group, mean proteinuria scores increased with time and the score was significantly higher at the end of treatment than at the start of treatment, indicating that lupus nephritis had progressed (Figures 4(b) and 4(c)). Mean proteinuria scores in the MT-1303-treated groups remained lower than those in the control group and were significantly lower at the end of administration in all MT-1303-treated groups than in the control group. In contrast, no significant difference in proteinuria scores was observed between the FK506-treated and control groups. In addition, urinary neutrophil gelatinase-associated lipocalin (NGAL), which is reported to be elevated in both acute and chronic kidney disease [27], was measured, and urinary NGAL level increased over time in the control group. On the other hand, urinary NGAL levels remained lower in the MT-1303 groups than in the control group ([Supplementary-material supplementary-material-1]).

Histological analysis of kidney sections was performed in half of the mice in each group except for the FK506 3 mg/kg group (Table 1). In MT-1303-treated groups, the severity of several histological changes including mesangial expansion, glomerular sclerosis, and interstitial infiltrates was lower than that in the control group, with MT-1303 at 0.3 mg/kg significantly reducing the severity of mesangial expansion and glomerular sclerosis. However, no significant changes were noted in perivascular infiltrates in MT-1303-treated groups. In the FK506 0.1 mg/kg group, while the scores for mesangial expansion and glomerular sclerosis were lower than those in the control group, there were no significant differences between the groups. These results indicate that MT-1303 significantly suppresses lupus nephritis progression in NZBWF1 mice and that MT-1303 is more potent than FK506.

### 3.5. MT-1303 Reduces Infiltration of T Cells into the Kidneys of NZBWF1 Mice

**Figure 5:**
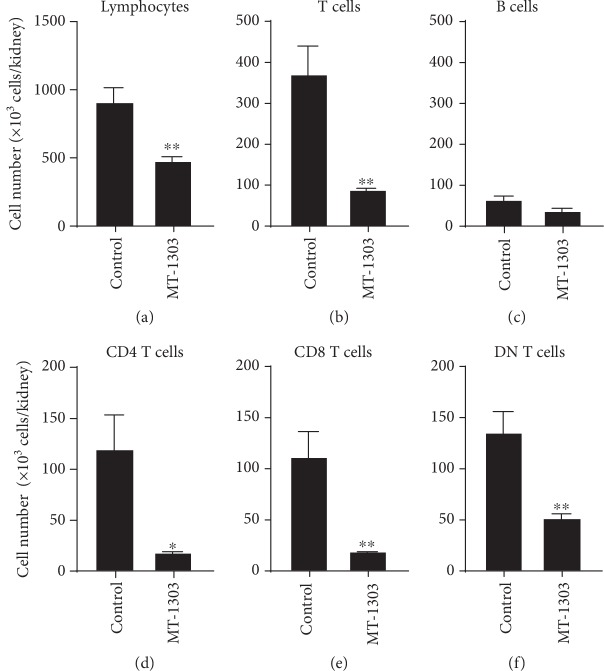
Effect of MT-1303 on the number of lymphocytes, T cells, B cells, CD4 T cells, and CD8 T cells in the kidneys of NZBWF1 mice. MT-1303 at 0.3 mg/kg was orally administered to NZBWF1 mice for 13 weeks from 28 weeks of age. The day after the final administration, the number of lymphocytes (a), T cells (b), B cells (c), CD4 T cells (d), CD8 T cells (e), and double-negative (DN) T cells (f) was determined by flow cytometry. Each column represents the mean ± S.E.M. cell count of 8 mice in each group. Statistical differences were calculated using Student's *t*-test (^∗^*p* < 0.05, ^∗∗^*p* < 0.01).

To elucidate the effects of MT-1303 on infiltration of T cells into the kidneys in NZBWF1 mice, kidneys were obtained from NZBWF1 mice administered with MT-1303 (0.3 mg/kg) or vehicle for 13 weeks until 41 weeks of age, and the lymphocytes that infiltrated into the kidneys were analyzed by flow cytometry. In the vehicle-treated control group, infiltration of lymphocytes including T cells and B cells into the kidneys was detected at 41 weeks (Figures 5(a)–5(f)). Meanwhile, treatment with MT-1303 significantly reduced the number of infiltrating T cells, CD4 T cells, CD8 T cells, and CD4^−^CD8^−^ double-negative (DN) T cells. Further, CD4 T cell and CD8 T cell counts decreased to less than 20% of those in the control group. Additionally, B cell counts in the MT-1303-treated group were lower than those in the control group, although there was no significant difference between the groups.

**Figure 6:**
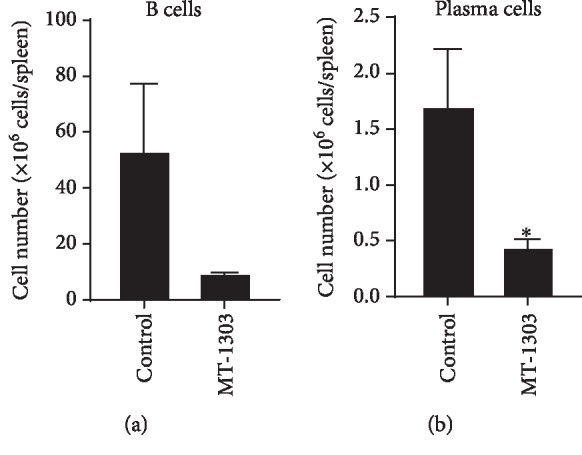
Effect of MT-1303 on the number of B cells and plasma cells in the spleen of NZBWF1 mice. MT-1303 at 0.3 mg/kg was orally administered to NZBWF1 mice for 13 weeks from 28 weeks of age. The spleen was obtained the day after the final administration. The number of B cells (a) and plasma cells (b) in the spleen was determined by flow cytometry. Results are expressed as the mean ± S.E.M. of 8 mice. Statistical differences were calculated using Student's *t*-test (^∗^*p* < 0.05).

In addition, we compared plasma cell counts in the spleen and serum levels of anti-dsDNA IgG in NZBWF1 mice after treatment for 13 weeks. The number of B cells and plasma cells in the spleen was significantly lower than that in the control group (Figures 6(a) and 6(b)); however, there was no significant difference in antibody levels between control and MT-1303-treated mice (data not shown).

## 4. Discussion

In the present study, oral administration of MT-1303 at 0.1 and 0.3 mg/kg was efficacious in MRL/*lpr* and NZBWF1 mice, two well-known murine SLE models that spontaneously develop lupus nephritis. MT-1303 not only inhibited the development of lupus nephritis when administered before onset but also improved the symptoms of lupus nephritis when administered after onset. Its efficacy in these models was superior or comparable to that of FK506, an already used treatment for lupus nephritis. Histological analysis additionally revealed that MT-1303 administration inhibited infiltration of T cells into the kidneys, mesangial expansion, and glomerular sclerosis.

Based on flow cytometric analysis, the number of T cells that infiltrated the kidneys increased in parallel with the progression of lupus nephritis in aged NZBWF1 mice. Immunohistochemical staining revealed T cell infiltration into the periglomeruli area in addition to the tubulointerstitium in MRL/*lpr* mice with lupus nephritis. Treatment with MT-1303 decreased the number of T cells infiltrating the kidneys and the number of lymphocytes in the blood. Th1 and Th17 cells reportedly mediate glomerulonephritis in MRL/*lpr* mice, and a deficiency in the chemokine receptor CXCR3 significantly improves morphology and function of nephritic kidneys via interference with the trafficking of both Th1 and Th17 cells into the inflamed kidneys [28]. In addition, DN T cells and CD4 T cells have been reported to infiltrate the kidneys of patients with lupus nephritis and produce the inflammatory cytokines interleukin-17 and interferon-*γ* [29]. We have reported that fingolimod, the first S1P_1_ receptor modulator, reduces infiltration of myelin antigen-specific Th17 and Th1 cells into the spinal cord [15]. Further, we have also confirmed that MT-1303 inhibits infiltration of colitogenic Th1 and Th17 cells into the colon in a mouse IBD model [30]. These results suggest that MT-1303 decreases infiltration of autoreactive Th1, Th17, and DN T cells into the kidneys by reducing the number of circulating lymphocytes in the blood, thereby eliciting a marked therapeutic effect on the progression of lupus nephritis in two SLE models.

In the mouse antibody response, fingolimod reportedly reduces the production of high-affinity, class-switched antibodies without affecting T cell-independent antibody production by reducing the formation of the germinal center in the T cell-dependent antibody formation system [18]. Additionally, treatment with fingolimod reduces the specific antibody production reaction against vaccination of anticipated novel antigens or recall antigens in MS patients [31]. In the present study, the effects of MT-1303 on blood anti-dsDNA antibody levels were contradictory. Administration of MT-1303 suppressed the increase in the number of plasma cells in the spleen of both strains, but the increase in anti-dsDNA antibody levels in the blood was suppressed only in MRL mice. However, these results are consistent with those observed with other S1P_1_ receptor modulators in MRL/*lpr* and NZBWF1 mice [19–22]. A multicenter, open-label phase Ib trial in patients with SLE has been conducted to evaluate the safety, pharmacodynamics, and exploratory efficacy of MT-1303, with 17 subjects administered with a low or high dose of MT-1303 for 24 weeks (ClinicalTrials.gov NCT02307643). Currently, this study has been completed, and the results are now undergoing finalization. Since the changes of biological markers in this clinical trial have been monitored, the results will reveal the effect of MT-1303 on anti-dsDNA antibody levels in SLE patients.

S1P_1_ receptor modulators have been developed for several autoimmune diseases, including MS, and MT-1303 has been evaluated in clinical trials for MS, psoriasis, Crohn's disease, and SLE. In a phase II study in more than 400 patients with RRMS, MT-1303 showed superior efficacy over the placebo control [3]. In inflammatory bowel disease (IBD), including ulcerative colitis (UC) and Crohn's disease (CD), there is some clinical evidence supporting the use of the S1P_1_ receptor modulators [17], and IBD is the next promising area of investigation after MS. Although the results of the phase II study of MT-1303 in CD patients were not as expected [32], we are developing MT-1303 for UC. In psoriasis, anti-IL-17 antibodies and the anti-IL-23p19 antibody are highly effective [33, 34], but the results of the phase II trial of MT-1303 were inferior to the effects of these antibodies (EudraCT number 2012-005750-27). On the other hand, although B cell-targeted belimumab is the only approved biological agent for SLE, its efficacy is limited [12], and a more effective agent with a novel mechanism of action is required. We expect that MT-1303, which affects trafficking of T and B cells, may have potential as a therapeutic agent for SLE; as noted above, a clinical trial for this condition has been completed and the results are now undergoing finalization.

Another S1P_1_ receptor modulator, cenerimod, has been evaluated in a phase II clinical trial in 64 patients with SLE (ClinicalTrials.gov NCT02472795). Although the results of this study have not yet been reported, a phase IIb study in 500 patients with active SLE (ClinicalTrials.gov NCT02472795) has been initiated. In the phase I clinical study of cenerimod, it was reported that heart rate decreased in healthy subjects after the initial administration from the lowest dose used in the SLE clinical trials [35]. MT-1303 has been reported to have no clinically significant bradyarrhythmia after the first dose at the anticipating clinical dose [2, 3], suggesting that MT-1303 may be superior to cenerimod in terms of cardiac effects, which is a concern with S1P_1_ receptor modulators.

## 5. Conclusions

Overall, this study demonstrates that MT-1303 shows superior or comparable therapeutic efficacy to FK506 in two SLE animal models. Moreover, it is likely that MT-1303 sequesters lymphocytes in secondary lymphoid tissues and the thymus and, thereby, not only inhibits infiltration of autoreactive T cells into inflammation sites but also affects the activation of autoreactive B cells and their differentiation to plasma cells by reducing the interaction between autoreactive T cells and B cells. Our nonclinical findings suggest that MT-1303 has the potential to be used as a therapeutic agent for patients suffering from SLE, including lupus nephritis.

## Figures and Tables

**Table 1:** Effect of MT-1303 and FK506 on histological scores in the kidneys.

Treatment	Histological score (mean ± S.E.M.)			
	Mesangial expansion	Glomerular sclerosis	Interstitial infiltrates	Perivascular infiltrates
Control	1.8 ± 0.5	1.3 ± 0.4	0.2 ± 0.2	1.0 ± 0.3
MT-1303 0.1 mg/kg	1.0 ± 0.5	0.7 ± 0.3	0.2 ± 0.2	1.2 ± 0.3
MT-1303 0.3 mg/kg	0.3 ± 0.3^∗^	0.2 ± 0.2^∗^	0.0 ± 0.0	1.5 ± 0.2
FK506 1 mg/kg	1.0 ± 0.5	0.8 ± 0.5	0.2 ± 0.2	1.3 ± 0.2

n = 6, Shirley-Williams test (^∗^*p* < 0.025, control group vs. MT-1303 groups), Wilcoxon test (control group vs. FK506 group).

## Data Availability

The data used to support the findings of this study are available from the corresponding author upon request.

## Supplementary Materials

Supplementary Materials Supplementary Figure 1: effects of MT-1303 and FK506 on urinary levels of NGAL in NZBWF1 mice.
