# Supplementary material for: Amiselimod (MT-1303), a Novel Sphingosine 1-Phosphate Receptor-1 Modulator, Potently Inhibits the Progression of Lupus Nephritis in Two Murine SLE Models
Source: J Immunol Res. 2019 Dec 23;2019:5821589. doi: 10.1155/2019/5821589 (PMC6942851; doi:10.1155/2019/5821589)
Supplement: Supplementary Materials — Supplementary Figure 1: effects of MT-1303 and FK506 on urinary levels of NGAL in NZBWF1 mice. [file 5821589.f1.docx]

**Supplementary Figure 1. Effects of MT-1303 and FK506 on urinary levels of NGAL in NZBWF1 mice.** MT-1303 and FK506 were orally administered to NZBWF1 mice daily from 30 to 40 weeks of age. Urinary NGAL concentrations were determined using mouse NGAL ELISA Kit (BioPorto Diagnostics, Gentofte, Denmark) once a week. Results were expressed as the mean ± S.E.M. of 12 mice in a logarithmic scale. Statistical significance was calculated using by the Williams test by comparison with the control group (*p<0.05).
